# Protective Effect of Caffeic Acid on Paclitaxel Induced Anti-Proliferation and Apoptosis of Lung Cancer Cells Involves NF-κB Pathway

**DOI:** 10.3390/ijms13056236

**Published:** 2012-05-21

**Authors:** Chien-Liang Lin, Ruei-Feng Chen, Jeff Yi-Fu Chen, Ying-Chieh Chu, Hui-Min Wang, Han-Lin Chou, Wei-Chiao Chang, Yao Fong, Wen-Tsan Chang, Chang-Yi Wu, Chien-Chih Chiu

**Affiliations:** 1 Department of Life Science and Institute of Zoology, National Taiwan University, Taipei 106, Taiwan; E-Mails: muscar6511@yahoo.com.tw (C.-L.L.); rfchen@ntu.edu.tw (R.-F.C.); 2 Department of Biotechnology, Kaohsiung Medical University, Kaohsiung 807, Taiwan; E-Mails: yifuc@kmu.edu.tw (J.Y.-F.C.); sillypig710@gmail.com (Y.-C.C.); d992050005@student.nsysu.edu.tw (H.-L.C.); 3 Department of Fragrance and Cosmetics Science, Kaohsiung Medical University, Kaohsiung 807, Taiwan; E-Mail: davidw@kmu.edu.tw; 4 Graduate Institute of Medical Genetics, Kaohsiung Medical University, Kaohsiung 807, Taiwan; E-Mail: weichiao.chang@gmail.com; 5 Chest Surgery, Chi-Mei Foundation Medical Center, Yung Kang City, Tainan 901, Taiwan; E-Mail: yaou.fong@msa.hinet.net; 6 Division of Hepatobiliary and Pancreatic Surgery, Department of Surgery, Kaohsiung Medical University Hospital, Kaohsiung 807, Taiwan; E-Mail: wtchang@kmu.edu.tw; 7 Department of Surgery, Faculty of Medicine, College of Medicine, Kaohsiung Medical University, Kaohsiung 807, Taiwan; 8 Department of Biological Sciences, National Sun Yat-Sen University, 70 Lien Hai Road, Kaohsiung 804, Taiwan; E-Mail: cywu@mail.nsysu.edu.tw

**Keywords:** caffeic acid, non-small cell lung cancer, paclitaxel, survivin, Bcl-2, NF-κB

## Abstract

Caffeic acid (CA), a natural phenolic compound, is abundant in medicinal plants. CA possesses multiple biological effects such as anti-bacterial and anti-cancer growth. CA was also reported to induce fore stomach and kidney tumors in a mouse model. Here we used two human lung cancer cell lines, A549 and H1299, to clarify the role of CA in cancer cell proliferation. The growth assay showed that CA moderately promoted the proliferation of the lung cancer cells. Furthermore, pre-treatment of CA rescues the proliferation inhibition induced by a sub-IC_50_ dose of paclitaxel (PTX), an anticancer drug. Western blot showed that CA up-regulated the pro-survival proteins survivin and Bcl-2, the down-stream targets of NF-κB. This is consistent with the observation that CA induced nuclear translocation of NF-κB p65. Our study suggested that the pro-survival effect of CA on PTX-treated lung cancer cells is mediated through a NF-κB signaling pathway. This may provide mechanistic insights into the chemoresistance of cancer calls.

## 1. Introduction

Lung cancer is the worldwide leading cause of death, and non-small cell lung cancer (NSCLC) accounts for 80% of total lung cancer cases [1]. Despite the fact that novel chemotherapeutic strategies against NSCLC have been developed, the treatments often fail in the majority of patients because of the insusceptibility of advanced lung cancer [2]. The rationale of most anti-tumor agents is to kill tumor cells by triggering apoptosis signaling pathways. Tumor cells may escape from apoptosis and become more chemoresistant. For example, the anti-cancer drug, paclitaxel (PTX), does not always completely induce apoptosis of tumor cells. Instead, tumor cells survive from apoptosis induced by PTX, and become more drug-resistant [3].

3,4-dihydroxy cinnamic acid or CA, a natural phenolic acid product, is found in fruits [4], wine [5,6], and coffee [7]. CA has been reported to exert diverse biological activities such as anti-bacterial [7], anti-oxidative [8] and anti-inflammatory [5]. In addition, previous studies demonstrated that CA sensitized multidrug-resistant human breast cancer cells towards doxorubicin treatment [9], and that CA induced apoptosis of cervical carcinoma cells [10]. A well-known CA derivative, caffeic acid phenethyl ester (CAPE), exhibits potent anti-cancer effects. Onori’s work reported that CAPE induced anti-proliferation and apoptosis of cholangiocarcinoma by inhibiting nuclear factor-κB (NF-κB) activation [11]. In comparison to the definite anti-cancer effect of CAPE, some studies showed that CA could exert protective effects. For example, CA was shown to prevent oxidative damage by enhancing antioxidant activity in cells [8]. Furthermore, CA significantly protects neurons of cerebellar granule [12] and human peripheral blood mononuclear cells [13] from apoptosis. Therefore, the actual effect of CA in chemoresistant cells remains unclear.

In the study, we investigated the effect of CA on proliferation and cell death of human lung cancer cells. Our results demonstrated the protective effect of CA on paclitaxel-induced cell death in lung cancer cells A549 and H1299, in which NF-κB was involved in the protective effect by CA.

## 2. Results and Discussion

### 2.1. Effect of CA on Proliferation of Lung Cancer Cells

Furthermore, CA was reported to protect liver cells from the attack of reactive oxygen species (ROS) [14]. In addition, CA showed protective effect against cytotoxic damages [14,15]. Although previous studies of CA have focused on its antioxidant and anti-inflammatory effects [16], little is known about the effect of CA on cancer treatment. Previous studies showed that CA induces tumor formation in the fore stomach and kidney in mice [17,18]. Moreover, it has been shown that the combination of CA and anti-cancer drugs reduces the cell growth of leukemic monocyte [19] and inhibits angiogenesis of renal carcinoma cells [20].

**Figure 1:**
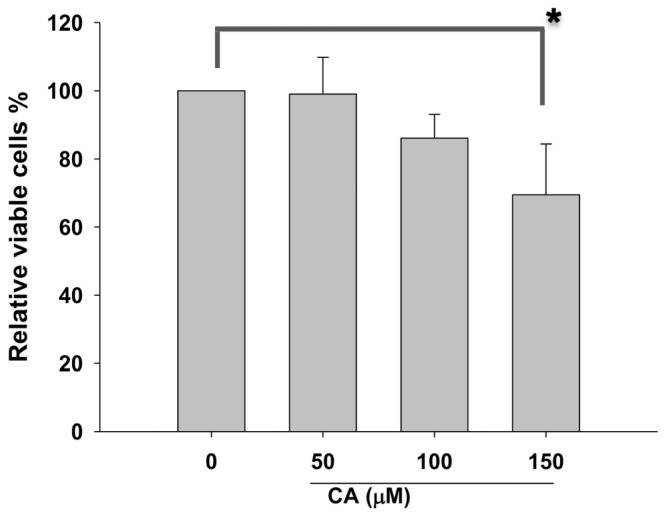
Proliferation of A549 cells with caffeic acid (CA) treatment. Cells were incubated with the indicated dose of CA (0, 50, 100, and 150 μM) for 48 h. The proliferation was determined by trypan blue exclusion assay. ^*^ *p* < 0.05 against vehicle control.

To examine the effects of CA on proliferation of NSCLC cells, the trypan blue based-proliferation assay was performed. As shown in Figure 1, CA exerts no significant cytotoxicity at the dose 50 and 100 μM towards NSCLC A549 cells. Our results showed that CA with a concentration lower than 100 μM has no significant cytotoxic effect on A549 cells

### 2.2. CA Rescues the PTX-Induced Anti-Proliferation in NSCLC Cells

**Figure 2:**
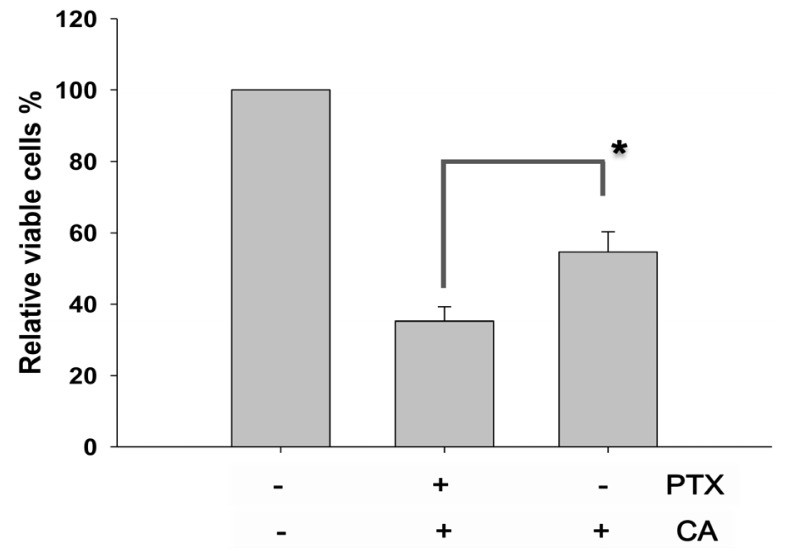
Protective effect of CA on paclitaxel (PTX)-treated A549 cells. Cells were co-treated with 100 μM of CA and the indicated drugs for 48 h respectively. Afterwards, the proliferation rate was determined using the assay of trypan blue exclusion. ^*^ *p* < 0.05 against PTX-treated alone.

To examine whether CA affects the anti-proliferation in lung cancer cells, PTX, a first-line drug against lung cancer was used in the study. In comparison with PTX treatment alone, the proliferation assay showed that 100 μM of CA pre-treatment significantly rescued the proliferation inhibition induced by PTX (Figure 2). Our results demonstrated the protective effect of CA on PTX-induced anti-proliferation in NSCLC cells.

### 2.3. CA Reduces the Activation of Pro-Apoptotic in NSCLC Cells

**Figure 3:**
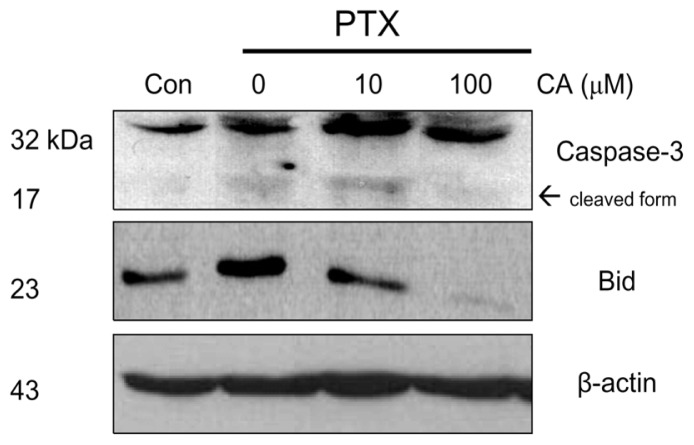
CA attenuates PTX-induced activation of pro-apoptotic proteins. A549 cells were pre-treated with the indicated dose of CA and PTX or PTX alone. Afterwards, cells were harvested and lysed. The whole cell lysates were analyzed using Western blot assay. Beta-actin was used as an internal control.

To determine whether CA protects PTX-induced apoptosis of A549 cells, we examined the protein levels of the cleaved caspase-3 and Bid by Western blotting. Our results found that PTX treated samples with 100 μM of CA pretreatment significantly reduced the level of cleaved caspase-3, a cytosolic indicator for apoptosis (Figure 3). Consistently, the protein level of pro-apoptotic factor Bid was decreased by CA, indicating the protective effect of CA on PTX-induced apoptosis of A549 cells.

Furthermore, the effect of CA was observed at 100 μM of CA but not at 10 μM, suggesting the protective effect of CA in a dose-responsive manner.

### 2.4. CA Up-Regulates the Protein Levels of Bcl-2 and Survivin

Survivin, a member of inhibitor of the apoptosis (IAP) family, and the pro-survival proteins Bcl-2 have been shown to be correlated with carcinogenesis, prognosis [21] and drug-resistance [22]. Numerous studies have demonstrated that down-regulation or inactivation of pro-survival proteins, including survivin and Bcl-2 [23], promotes anti-cancer efficacy or sensitizes tumor cells toward chemotherapeutic drugs in human tumor cells [24].

**Figure 4:**
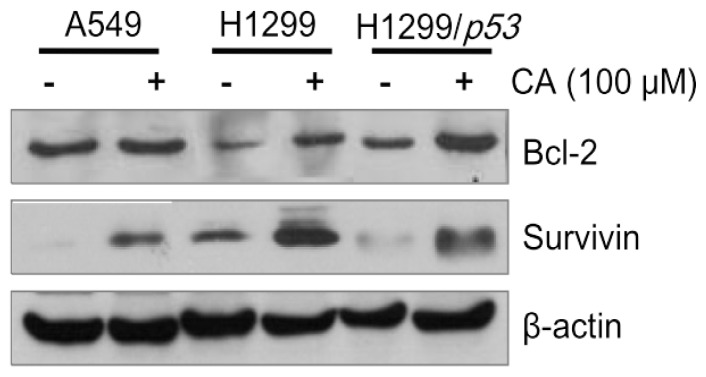
The modulations of pro-survival proteins by CA. The non-small cell lung cancer (NSCLC) cell lines A549, H1299 and H1299 transfected with functional p53 were treated with 100 μM CA for 24 h, respectively. Afterwards, the protein levels of pro-survival Bcl-2 and survivin were determined using Western blot assay. Beta-actin was used as an internal control.

Next, in order to examine how CA attenuates PTX-induced apoptosis of lung cancer cells, Western blot for detecting Bcl-2 and survivin was performed (Figure 4). As shown in Figure 4, the results showed that pre-treatment of CA induced the accumulation of pro-survival proteins Bcl-2 and survivin in A549 (wt-*p53*) and H1299 (null-*p53*). The anti-apoptotic activity of CA has been previously reported to be modulated through a Bcl-2-independent pathway [13]. On the contrary, our result showed that the CA mediated anti-apoptosis in lung cancer (Figure 4). We also observed that the protein level of survivin in H1299 cells was higher than that in A549 cells after CA pre-treatment. Moreover, CA-induced up-regulation of survivin was attenuated in H1299 cells with the ectopically expressed wild-type *p*53 (Figure 4), indicating the antagonistic role of p53 in the protective effect of CA.

Furthermore, CA seems to induce higher levels of survivin and Bcl-2 proteins in H1299 cells, which carry non-functional p53 than in A549 cells that carry functional p53. Interestingly, CA-induced expression of pro-survival survivin and Bcl-2 proteins in p53-expressing H1299 cells was attenuated compared to the parental cells (Figure 4). Accordingly, we suggest that p53 may antagonize the protective effect of CA on PTX-induced anti-proliferation and apoptosis of lung cancer cells.

### 2.5. CA Induces Up-Regulation and Activation of NF-κB

The transcription factor NF-κB modulates genes involved in cell survival, proliferation and apoptosis [25]. The NF-κB p65 is the most abundant isoform of NF-κB in cells [1]. Inactivated NF-κB forms a heterodimer with an endogenous inhibitor IκB in the cytoplasm [25]. With physiological stimuli or stresses, IκB kinases (IκK) phosphorylate the IκB protein, resulting in the dissociation of NF-κB from IκB [25]. The activated NF-κB translocates into the nucleus and turns on its target genes [25]. Accumulating evidence has shown that overexpression or constitutive activation of NF-κB is common in many cancer cells, including lymphoma [26], lung cancer cells [27] and liver cancer [1]. Other reports indicate that NF-κB plays a role in chemoresistance of breast cancer [28]. Furthermore, it is well known that the expression of anti-apoptotic Bcl-2 genes [25–27], and the member of inhibitor of apoptosis (IAPs) survivin is the down-stream target gene of NF-κB [29].

**Figure 5:**
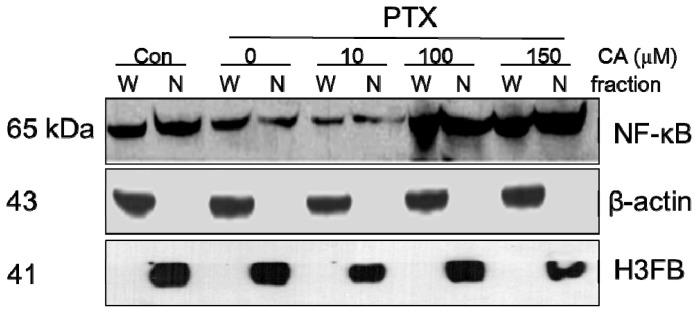
CA modulates the expression and nuclear translocation of NF-κB p65. A549 cells were pre-treated with the indicated dose of CA and PTX or PTX alone. The nuclear and cytosol extracts were analyzed using Western blot assay. W = whole cell lysate; N = nuclear fraction. β-actin was used as an internal control of cytosol and H3 histone, family 3B (H3FB) as an internal control of the nucleus.

To determine the role of NF-κB p65 in CA-induced protective effect, the nuclear/cytosolic fractions of proteins were isolated for Western blot analysis. The results showed that PTX decreased the level of NF-κB p65 protein both in the cytosol and the nucleus. On the contrary, pre-treatment of CA increased the total protein and nuclear extract of NF-κB p65 in A549 cells significantly. These results also indicated that higher doses of CA (≥100 μM) caused nuclear translocation of NF-κB p65 (Figure 5).

The transcript factor NF-κB, with anti-apoptosis ability [13,22], was found to be overexpressed in numerous multidrug-resistant cancer cells [30–33]. Our results demonstrated that the increased level of Bcl-2 and survivin by CA pre-treatment correlated with the activity of NF-κB (Figure 4). As expected, we demonstrated that PTX induced cytosolic accumulation of NF-κB in A549 cells (Figure 5). However, pre-treatment of CA significantly increased the nuclear abundance of NF-κB in PTX-treatment A549 cells at the dose of 100 μM PTX but not at the dose of 10 μM. These above results not only suggested that the protective effect of CA might involve the activation of the NF-κB p65 pathway, but that the CA-induced activation of NF-κB was dose-responsive.

## 3. Experimental Section

### 3.1. Cell Cultures

Human non-small cell lung cancer (NSCLC) cell lines, A549 (wild type-*p53*), H1299 (null-*p53*) as well as H1299 cells transfected with the functional wild-type *p53* were cultured in Dulbecco’s modified Eagle’s medium (DMEM) (Gibco, Grand Island, NY, USA) supplemented with 10% fetal bovine serum (FBS), 100 units/mL penicillin, 100 μg/mL streptomycin, 0.03% glutamine, and 1 mM sodium pyruvate. Cells were incubated at 37 °C in a humidified atmosphere containing 5% CO_2_.

### 3.2. Reagents and Antibodies

Paclitaxel (PTX), and Caffeic acid (3,4-dihydroxycinnamic acid, CA) were purchased from Sigma-Aldrich (St. Louis, MO, USA). Antibodies against survivin, Bcl-2, survivin, NF-κB p65 and β-actin (Santa Cruz Biotechnology, Santa Cruz, CA, USA), and antibodies against H3 histone, family 3B (H3FB) were purchased from Abnova.

### 3.3. Growth Proliferation Test

Cell growth was determined by the trypan blue dye exclusion assay as described previously [33]. In brief, cells were treated with vehicle control (DMSO), PTX alone or pre-treated with CA for 24 h prior to PTX treatment, respectively. After incubation, cells were exposed to 0.2% trypan blue, then counted by the Countess™ automated cell counter (Invitrogen, Carlsbad, CA, USA).

### 3.4. Western Blot Analysis

The Western blot assay was described previously [1]. Briefly, cells were harvested and lysed. Lysates were centrifuged and the protein concentration was determined. Then 40 μg of protein lysate was separated by SDS-polyacrylamide gel electrophoresis (SDS-PAGE) and electrotransferred. Afterwards, the membrane was blocked with 5% non-fat milk (Sigma-Aldrich), followed by incubation with primary and secondary antibodies against specific proteins and corresponding antibodies, respectively. The chemiluminescence signals were detected by ECL detection kit (Amersham Piscataway, NJ, USA).

### 3.5. Preparation of Cytosolic and Nuclear Extracts

The preparation of cytoplasmic and nuclear extracts was performed using the Nuclear Extract Kit (Active Motif, Carlsbad, CA, USA) according to the manual of the manufacturer. In brief, 1 × 10^7^ of CA-treated A549 cells were washed with 1 mL ice-cold PBS/phosphatase inhibitor, lysed in hypotonic buffer, and then centrifuged at 14,000 *g* for 30 s at 4 °C. The supernatant was transferred into fresh 1.5 microtubes as cytoplasmic fraction, then pellets were resuspended in 50 μL complete lysis buffer, and centrifuged at 14,000 *g* for 10 min at 4 °C, supernatants (nuclear fraction) were saved and divided into aliquots. The protein concentrations were quantified and analyzed by Western blot assay.

### 3.6. Statistical Analysis

All data were presented as means ± S.D. The Student’s t-test analysis was used to test the mean difference between two groups.

## 4. Conclusions

**Figure 6:**
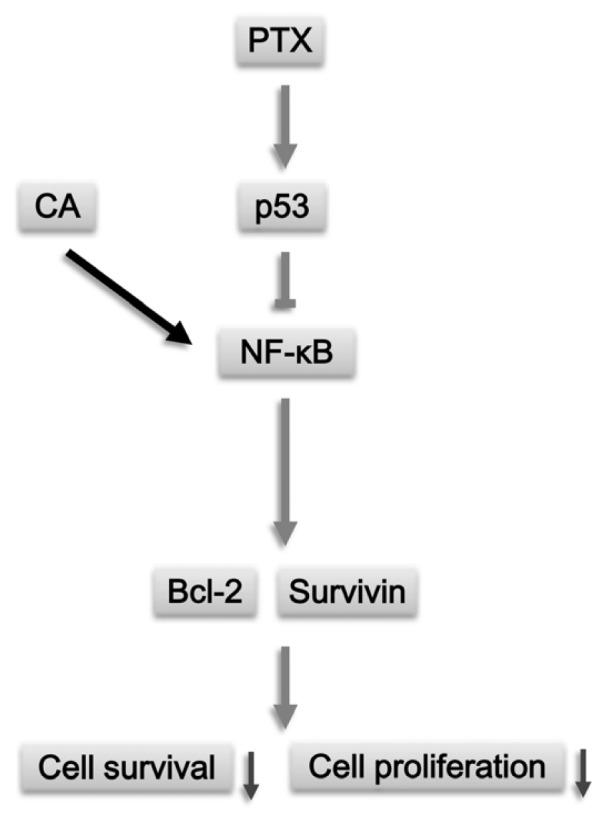
Proposed pathways of CA-mediated protective effect on PTX-induced proliferative inhibition in NSCLC cells. PTX is well-known for inducing the disturbance of mitosis, and PTX inhibited the proliferation of NSCLC cells in the study. Pre-treatment of CA facilitates the increased level and nuclear translocation of transcription factor NF-κB. The activation of NF-κB up-regulates the expression of its downstream genes Bcl-2 and survivin. On the contrary, p53 may antagonize the protective effect of NF-κB, and therefore attenuates the expression of Bcl-2 and survivin. Eventually, the increased proteins of Bcl-2 and survivin play protective roles in PTX-treated cancer cells, and attenuate the anti-proliferation effect of PTX on lung cancer cells.

Our present study demonstrated the protective effect of CA on PTX-induced anti-proliferation in NSCLC cells. The protective effect of CA is, at least partly, mediated by the NF-κB pathway (Figure 6). Furthermore, the protective effect of CA was dose-dependent and depended on cell types. Additionally, *p53* might act as an antagonist toward the protective effect of CA directly or indirectly. In conclusion, our results may shed light on the role of CA in chemotherapy, such as PTX-treatment, and this finding could give an important index for PTX-based chemotherapeutics.

## References

[1] Chiu C.C., Chen J.Y., Lin K.L., Huang C.J., Lee J.C., Chen B.H., Chen W.Y., Lo Y.H., Chen Y.L., Tseng C.H. (2010). p38 MAPK and NF-κB pathways are involved in naphtho[1,2-b] furan-4,5-dione induced anti-proliferation and apoptosis of human hepatoma cells. Cancer Lett.

[2] Li S., Bao P., Li Z., Ouyang H., Wu C., Qian G. (2009). Inhibition of proliferation and apoptosis induced by a Na^+^/H^+^ exchanger-1 (NHE-1) antisense gene on drug-resistant human small cell lung cancer cells. Oncol. Rep.

[3] Tabuchi Y., Matsuoka J., Gunduz M., Imada T., Ono R., Ito M., Motoki T., Yamatsuji T., Shirakawa Y., Takaoka M. (2009). Resistance to paclitaxel therapy is related with Bcl-2 expression through an estrogen receptor mediated pathway in breast cancer. Int. J. Oncol.

[4] Hsu F.L., Chen Y.C., Cheng J.T. (2000). Caffeic acid as active principle from the fruit of Xanthium strumarium to lower plasma glucose in diabetic rats. Planta Med.

[5] Giovannini L., Migliori M., Filippi C., Origlia N., Panichi V., Falchi M., Bertelli A.A., Bertelli A. (2002). Inhibitory activity of the white wine compounds, tyrosol and caffeic acid, on lipopolysaccharide-induced tumor necrosis factor-alpha release in human peripheral blood mononuclear cells. Int. J. Tissue React.

[6] Simonetti P., Gardana C., Pietta P. (2001). Caffeic acid as biomarker of red wine intake. Methods Enzymol.

[7] Almeida A.A., Farah A., Silva D.A., Nunan E.A., Gloria M.B. (2006). Antibacterial activity of coffee extracts and selected coffee chemical compounds against enterobacteria. J. Agric. Food Chem.

[8] Chung M.J., Walker P.A., Hogstrand C. (2006). Dietary phenolic antioxidants, caffeic acid and Trolox, protect rainbow trout gill cells from nitric oxide-induced apoptosis. Aquat. Toxicol.

[9] Ahn C.H., Choi W.C., Kong J.Y. (1997). Chemosensitizing activity of caffeic acid in multidrug-resistant MCF-7/Dox human breast carcinoma cells. Anticancer Res.

[10] Chang W.C., Hsieh C.H., Hsiao M.W., Lin W.C., Hung Y.C., Ye J.C. (2010). Caffeic acid induces apoptosis in human cervical cancer cells through the mitochondrial pathway. Taiwan J. Obstet. Gynecol.

[11] Onori P., DeMorrow S., Gaudio E., Franchitto A., Mancinelli R., Venter J., Kopriva S., Ueno Y., Alvaro D., Savage J. (2009). Caffeic acid phenethyl ester decreases cholangiocarcinoma growth by inhibition of NF-κB and induction of apoptosis. Int. J. Cancer.

[12] Tian X.F., Pu X.P. (2004). Caffeic acid (CA) protects cerebellar granule neurons (CGNs) from apoptosis induced by neurotoxin 1-methyl-4-phenylpyridnium (MPP+). Beijing Da Xue Xue Bao.

[13] Khanduja K.L., Avti P.K., Kumar S., Mittal N., Sohi K.K., Pathak C.M. (2006). Anti-apoptotic activity of caffeic acid, ellagic acid and ferulic acid in normal human peripheral blood mononuclear cells: A Bcl-2 independent mechanism. Biochim. Biophys. Acta.

[14] Lima C.F., Fernandes-Ferreira M., Pereira-Wilson C. (2006). Phenolic compounds protect HepG2 cells from oxidative damage: Relevance of glutathione levels. Life Sci.

[15] Kang K.A., Lee K.H., Zhang R., Piao M., Chae S., Kim K.N., Jeon Y.J., Park D.B., You H.J., Kim J.S. (2006). Caffeic acid protects hydrogen peroxide induced cell damage in WI-38 human lung fibroblast cells. Biol. Pharm. Bull.

[16] Mirzoeva O.K., Calder P.C. (1996). The effect of propolis and its components on eicosanoid production during the inflammatory response. Prostaglandins Leukot. Essent. Fatty Acids.

[17] Lutz U., Lugli S., Bitsch A., Schlatter J., Lutz W.K. (1997). Dose response for the stimulation of cell division by caffeic acid in forestomach and kidney of the male F344 rat. Fundam. Appl. Toxicol.

[18] Hagiwara A., Hirose M., Takahashi S., Ogawa K., Shirai T., Ito N. (1991). Forestomach and kidney carcinogenicity of caffeic acid in F344 rats and C57BL/6N x C3H/HeN F1 mice. Cancer Res.

[19] Nardini M., Leonardi F., Scaccini C., Virgili F. (2001). Modulation of ceramide-induced NF-κB binding activity and apoptotic response by caffeic acid in U937 cells: Comparison with other antioxidants. Free Radic. Biol. Med.

[20] Jung J.E., Kim H.S., Lee C.S., Park D.H., Kim Y.N., Lee M.J., Lee J.W., Park J.W., Kim M.S., Ye S.K. (2007). Caffeic acid and its synthetic derivative CADPE suppress tumor angiogenesis by blocking STAT3-mediated VEGF expression in human renal carcinoma cells. Carcinogenesis.

[21] Cohen C., Lohmann C.M., Cotsonis G., Lawson D., Santoianni R. (2003). Survivin expression in ovarian carcinoma: Correlation with apoptotic markers and prognosis. Mod. Pathol.

[22] Zaffaroni N., Pennati M., Colella G., Perego P., Supino R., Gatti L., Pilotti S., Zunino F., Daidone M.G. (2002). Expression of the anti-apoptotic gene survivin correlates with taxol resistance in human ovarian cancer. Cell. Mol. Life Sci.

[23] Ramnath V., Rekha P.S., Kuttan G., Kuttan R. (2009). Regulation of caspase-3 and Bcl-2 expression in Dalton’s lymphoma ascites cells by Abrin. Evid.-Based Complement. Alternat. Med.

[24] Raviv Z., Zilberberg A., Cohen S., Reischer-Pelech D., Horrix C., Berger M.R., Rosin-Arbesfeld R., Flescher E. (2011). Methyl jasmonate down-regulates survivin expression and sensitizes colon carcinoma cells towards TRAIL-induced cytotoxicity. Br. J. Pharmacol.

[25] Ghosh S., Karin M. (2002). Missing pieces in the NF-κB puzzle. Cell.

[26] Heckman C.A., Mehew J.W., Boxer L.M. (2002). NF-κB activates Bcl-2 expression in t(14;18) lymphoma cells. Oncogene.

[27] Tang X., Liu D., Shishodia S., Ozburn N., Behrens C., Lee J.J., Hong W.K., Aggarwal B.B., Wistuba I.I. (2006). Nuclear factorκB (NFκB) is frequently expressed in lung cancer and preneoplastic lesions. Cancer.

[28] Weldon C.B., Burow M.E., Rolfe K.W., Clayton J.L., Jaffe B.M., Beckman B.S. (2001). NF-κB-mediated chemoresistance in breast cancer cells. Surgery.

[29] Karin M., Lin A. (2002). NF-κB at the crossroads of life and death. Nat. Immunol.

[30] Ding R.K., Ding W., Wang C.K., Lu H., He Z.M. (2008). The effect of survivin on multidrug resistance by epidermal growth factor receptor 2 via NF-κB activating in breast cancer cells. Cell Biol. Int.

[31] Zhang X.H., Su L.D., Lu Q.H., Liang Y., Zhao X.Y. (2004). Relationship between drug resistance and the expression of NF-κB induced in leukemic cells. Zhejiang Da Xue Xue Bao Yi Xue Ban.

[32] Salvatore C., Camarda G., Maggi C.A., Goso C., Manzini S., Binaschi M. (2005). NF-κB activation contributes to anthracycline resistance pathway in human ovarian carcinoma cell line A2780. Int. J. Oncol.

[33] Chiu C.C., Liu P.L., Huang K.J., Wang H.M., Chang K.F., Chou C.K., Chang F.R., Chong I.W., Fang K., Chen J.S. (2011). Goniothalamin inhibits growth of human lung cancer cells through DNA damage, apoptosis, and reduced migration ability. J. Agric. Food Chem.

